# The Impact of Organisational Changes on Teamwork in Healthcare: A Systematic Review

**DOI:** 10.21315/mjms-01-2025-074

**Published:** 2025-08-30

**Authors:** Fathiah Jabir, Mohamad Izzi Zahari, Faiz Daud

**Affiliations:** Department of Public Health Medicine, Faculty of Medicine, Universiti Kebangsaan Malaysia, Kuala Lumpur, Malaysia

**Keywords:** organisational behaviour, organisational development, hospital, clinician, health managers

## Abstract

Healthcare organisations have to frequently undergo significant reforms, ranging from structural reforms to operational modifications, to address evolving challenges, such as an ageing population, technological advancements, and emerging diseases. These organisational changes significantly affect teamwork in healthcare, which is a critical component for ensuring patient safety, enhancing clinical performance, and maintaining staff satisfaction. The relationship between organisational changes and teamwork is ill-defined. PubMed, Scopus, ProQuest, and Web of Science (WOS) databases were searched for relevant literature on the impact of organisational changes on teamwork processes and outcomes in healthcare settings. Based on predefined selection criteria, 24 studies were considered suitable for inclusion; data from these studies were synthesised using thematic analysis. Three primary themes of organisational changes—organisational restructuring, operational changes, and adaptive response and capacity development—which affected team dynamics in various ways, were identified. Both planned and emergent changes produce mixed effects on teamwork; while planned changes are aimed at improving cooperation and efficiency and minimising negative impacts, they may inadvertently disrupt teamwork. In contrast, emergent changes can encourage resilience and adaptability within teams. Finally, the success of organisational changes in improving teamwork outcomes depends heavily on implementation strategies, contextual factors, and team characteristics. This review highlights the importance of employing established change management frameworks to address the complexities of organisational change. Further research, particularly from low- and middle-income countries, is essential to develop strategies and approaches for establishing effective healthcare teams in diverse settings.

## Introduction

Healthcare organisations around the world need to adapt constantly to evolving demands. Increasing life expectancy, coupled with an ageing population possessing a broad spectrum of health statuses, poses a significant challenge to healthcare providers and professionals (1). Simultaneously, newly emerging and recurring diseases, such as the recent COVID-19 pandemic and the Mpox outbreak, have put additional pressure on the healthcare system. To address these healthcare challenges while incorporating the latest technological advancements as well as policy reforms, healthcare organisations need to relentlessly revise their service delivery model that can accommodate multifaceted amendments ranging from structural reorganisations to organisational development initiatives that enhance workforce capacities and organisational culture (2, 3). As healthcare delivery becomes increasingly complex, the efficacy of these organisational reforms largely depends on the adaptability and functionality of existing healthcare teams within the new structure and arrangements.

Teamwork is essential in delivering quality healthcare services; having a good relationship with team members who could act as support in times of need helps ensure patient safety, clinical performance, healthcare worker job satisfaction (4–6), and a good work-life balance (7). A variety of factors, such as clear leadership, effective communication, and trust, may determine team effectiveness (8); however, organisational changes can significantly impact team dynamics, potentially affecting both team processes and outcomes. While several studies have independently examined the effects of organisational changes in healthcare and the effectiveness of teamwork, there is a lack of understanding regarding the influence of organisational changes on healthcare teams (3, 5, 9). The relationship between organisational changes and teamwork is particularly complex because the same changes may produce different outcomes across different healthcare settings, which is further complicated by the distinction between the nature of the change introduced.

Accordingly, this systematic review aimed to synthesise the available evidence on organisational changes in healthcare settings and evaluate their impact on teamwork. Specifically, it seeks to address two research questions: i) what are the types of organisational changes occurring in healthcare settings?; and ii) how do these changes impact teamwork processes and outcomes? Understanding these relationships is important, especially for healthcare administrators and policymakers, to ensure the efficient implementation of organisational changes while maintaining or enhancing team effectiveness.

## Methods

### Search Strategy

This systematic review was conducted in accordance with the Preferred Reporting Items for Systematic Reviews and Meta-analyses (PRISMA) guidelines of 2020 (10). The PubMed, Scopus, ProQuest, and Web of Science (WOS) databases were searched in November 2024 for relevant publications using predefined keywords and synonyms related to organisational changes, teamwork, and healthcare. The search strings used for the literature search are provided in Supplementary Table A.

### Eligibility Criteria

Only peer-reviewed empirical or original studies employing either qualitative, quantitative, or mixed-methods designs were included; reviews or abstracts from conference proceedings were excluded. No restrictions were imposed regarding language or the year of study implementation. The following criteria were used for study selection:

- Population: Healthcare teams across various settings. Studies involving undergraduate students were excluded, as they are not considered healthcare workers (HCW) yet.
- Exposure: Organisational changes (the alteration of operations within an institution), such as restructuring, operational changes, or adaptive responses.
- Outcomes: Any teamwork-related outcomes were considered. Studies lacking specific outcomes on teamwork processes or results were excluded.

### Screening

Two reviewers independently performed an initial screening of the study titles and abstracts using the aforementioned eligibility criteria. Subsequently, the full texts of potentially eligible studies were meticulously reviewed to determine their suitability for inclusion. In case of disagreements, a third reviewer was consulted for resolution.

### Data Extraction

After ascertaining eligibility, both reviewers independently performed data extraction using a pre-determined template, which was created through discussion between the authors. A third reviewer was approached to resolve any disagreements. The following data were recorded for each study: study characteristics [year of publication, author(s), country, study design, and sampled population], organisational changes, and reported impacts on teamwork. These data were extracted to answer the research questions posed earlier.

### Quality Assessment

The mixed-methods appraisal tool (MMAT) 2018 (11) was used to assess the quality of the included studies. The overall quality score was presented using stars (*) ranging from one star to five stars: * for a score of 1/5 (least quality); ** for a score of 2/5; *** for a score of 3/5; **** for a score of 4/5; and ***** for a score of 5/5 (highest quality). Typically, 15 criteria (instead of five) are used to rate mixed-methods studies, the rationale being that the overall quality of a combination cannot exceed the quality of its weakest component. Thus, the overall quality score of a study was the lowest for each study component. Two independent reviewers assessed all the included studies and assigned an overall quality score to each study. Any disagreement regarding the quality appraisal was discussed prior to engaging a third reviewer for resolution, if needed.

### Data Synthesis

In this review, a qualitative synthesis was used to enable iterative comparisons across the primary data sources (12). Thematic analysis was employed to qualitatively synthesise the data extracted from the included studies and formulate themes based on the patterns identified. Similar or related extracted data were aggregated into groups, and data were re-examined to identify potential subthemes within the main themes. Throughout the process, the reviewers engaged in active discussions to reach consensus in case of inconsistencies, interpretations, or ideas associated with the data analysis.

## Results

### Characteristics of the Included Studies

**Figure 1:**
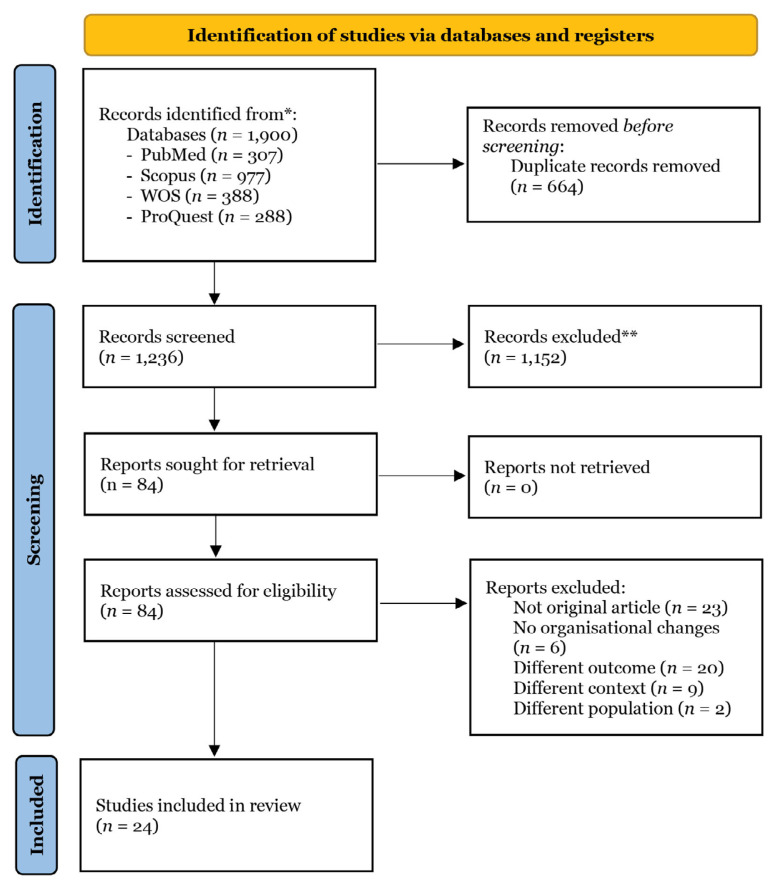
PRISMA flowchart for articles’ selection

The search strategy yielded a total of 1,236 records; following the screening process, 24 articles were deemed eligible for inclusion in the review. The screening process is outlined in Figure 1, and a descriptive analysis of the 24 studies is shown in Table 1.

Twenty-three out of the 24 studies were conducted in high-income countries; one study was from South Africa (13), which is an upper-middle-income country. Most studies (*n* = 11; 45.8%) employed qualitative research designs (14–24), whereas six studies (25.0%) (13, 25–29) used mixed-method research designs. The included studies were published from 2003 onwards, with the majority (*n* = 18; 75.0%) published in the past 10 years (15–19, 21–24, 26–34). Nine studies (37.5%) were conducted among primary care workers (13, 14, 18–20, 27, 29, 33, 34), three (12.5%) among HCW in the intensive care unit (ICU) (22, 30, 35), two (8.3%) among workers in an emergency department (28, 32), and the remaining were conducted at different health services and facilities. Only one study was conducted across the entire health system of a country (16).

### Quality Assessment of Included Studies

Overall, 12 studies received a rating of ***** (14, 15, 16–24, 33), 10 received a rating of **** (13, 26–32, 34, 35), and the remaining two articles were graded *** (36) and ** (25), respectively (Table 2). All qualitative studies received a rating of *****; the majority of these studies provided detailed descriptions of their data collection methods and clearly derived findings from the data, often using quotations to support the identified themes. The overall quality of studies employing other study designs was also high, since they employed adequate sampling strategies to ensure representativeness. However, the risk of nonresponse bias was generally high due to relatively low response rates in many of the included studies. Several mixed-method studies effectively integrated different data types; however, they did not identify or address discrepancies and inconsistencies between qualitative and quantitative results.

### Organisational Changes

To answer the first research question, three key themes related to organisational changes were identified from the included studies: i) organisational restructuring; ii) operational changes; and iii) adaptive response and capacity development; each theme comprised its own three subthemes (Table 3). The three themes and the associated subthemes are discussed further.

#### Organisational Restructuring

This theme encompasses the underlying changes in the structural design of healthcare services, including the organisation, staffing, and physical arrangement of such services. This theme further incorporated the following subthemes: service integration and merger; workforce reorganisation; and physical and environmental changes.

The subtheme of service integration and merger embodies the changes in an organisation’s structure through services or unit consolidation, hospital amalgamation, mergers of different wards or departments, and integration of care services. Gulliver et al. (36) described the effects of integrating mental health and social care services, wherein staff from separate organisations were merged into a single trust. Likewise, Vifladt et al. (30) reported on a department-level merger in which three of six Norwegian hospitals merged their general and medical ICUs into mixed ICUs. On a larger scale, Allen et al. (25) documented the amalgamation of two hospitals, causing further changes in staffing and structure.

The second subtheme, workforce reorganisation, is also frequently described as part of organisational changes. Rodriquez (22) documented the process of implementing workforce reductions and role combinations driven by cost-cutting measures. Notably, Huby et al. (20) described the creation of elite multidisciplinary groups that fundamentally changed team structure and management.

The third subtheme of organisational restructuring, physical and environmental changes, involves redesigning physical spaces to optimise teamwork or facility relocations (27, 28, 32). Mash et al. (13) noted that the colocation of multidisciplinary teams aimed at improving collaboration requires careful attention to spatial arrangements.

#### Operational Changes

This theme highlights the changes occurring at the level of work execution, management, or coordination within a healthcare organisation. The three subthemes emerging during the thematic analysis were process and technology changes, governance and management changes, and changes in working methods and protocols.

Process and technology changes include workflow modifications, implementation of new digital and communication systems, and care delivery changes. Bentley et al. (27) documented the introduction of electronic health records, while Rogers et al. (16) discussed the effects of a technological shift to using virtual meetings during the COVID-19 pandemic.

The second subtheme, governance and management changes, is related to the modifications to leadership and decision-making processes in healthcare teams. Mash et al. (13) highlighted the significance of adopting participative leadership approaches aimed at engaging team members through open dialogue and a shared vision. Other studies included in this subtheme described organisational changes in decision-making structures and new management structures (20, 22, 36).

Finally, under the changes in working methods and protocols subtheme, the included studies covered changes in standard procedures, care protocols, quality systems, and work standardisation (15, 28, 29, 34). For instance, MacKinnon et al. (15) described the implementation of structured communication tools and team huddles as part of the introduction of a new care delivery model.

#### Adaptive Response and Capacity Development

The third theme in organisational changes focuses on enhancing the organisation’s ability to respond to challenges and improve team functioning via capacity building and changing the organisational or team culture. Under this theme, three subthemes were identified: capacity and skills enhancement; collaborative practice and culture; and response and adaptation to external pressure.

The first subtheme, capacity and skills enhancement, implicates the importance of implementing training programmes and carrying out simulation exercises for both professional development and team competencies. For instance, Ballangrud et al. (17) described the implementation of the Team Strategies and Tools to Enhance Performance and Patient Safety (TeamSTEPPS) longitudinal training programme to enhance interprofessional teamwork.

The second subtheme, collaborative practice and culture development, emerged from the analysis through various interventions. Maun et al. (19) stated that interdisciplinary collaboration can be facilitated through regular team meetings, while Mash et al. (13) highlighted the importance of establishing a shared vision among team members.

The final subtheme, response and adaptation to external pressure, became particularly relevant as organisations face various demands. Cain (23) described how healthcare teams adapted to Medicare policy changes, while Pulido-Fuentes et al. (18) and Rogers et al. (16) reported rapid adaptations to challenges emerging during the COVID-19 pandemic.

### Impact of Organisational Changes on Team and Teamwork

For the second research question, thematic analysis of how organisational changes affect teamwork revealed two distinct aspects—team processes and team outcomes—each with its subthemes (Table 4).

#### Team Processes

Team processes encompass operational changes in the functioning of healthcare teams. Under this aspect, two subthemes were identified on further analysis. The first subtheme was leadership and decision-making, which explores the organisational changes leading to authority distribution. These changes were demonstrated well in the study by Huby et al. (20), where decision-making was concentrated in fewer hands, namely the “elite” multidisciplinary groups, who were tasked to monitor and control their colleagues’ behaviour. Other observed changes included how a team is led or coordinated; for instance, the adoption of a participative leadership style, coupled with the establishment of semi-autonomous practice teams comprising physicians and nurses, helped to reorient practices involving more open dialogue (13).

The second subtheme was identified as “communication and task organisation”; this subtheme uncovers how teams change their ways of communication and reorganise work within the team. MacKinnon et al. (15) introduced the Care Delivery Model Redesign aimed at communication and task organisation; the model was based on structured communication tools and involved changing task distribution between registered nurses (RNs) and licensed practical nurses (LPNs), requiring continuous negotiation for assigning patients based on acuity and stability.

#### Team Outcomes

Among the 24 included studies, two subthemes related to team outcomes were identified, namely, team performance and impact on team members. The impact of organisational changes on the efficacy of team functionality yielded mixed results. A study reported that some teams experienced decreased collaboration, weaker cooperation, and poorer teamwork culture due to the splitting of established teams (30). In contrast, another study described improved evaluations of collaboration, work relationships, and service quality by the staff as a result of team reorganisation (31); however, the implemented changes substantially impacted staff well-being and retention, with evidence of increased emotional exhaustion and diminished job satisfaction, particularly during the initial phases of implementation (36). Furthermore, healthcare personnel experienced strained interpersonal relationships and a sense of diminished agency (21), resulting in some individuals departing from their respective teams (19). Another study reported varying long-term viability of these changes, with certain teams exhibiting initial improvements in collaborative efforts, followed by a subsequent decline over time (18).

### Planned and Emergent Changes

In addition to the key research questions, this review examined whether the aforementioned organisational changes were planned or emergent, based on the description provided in each article. In most studies, the changes described were primarily planned (*n* = 19; 79.2%); two studies primarily reported implementing emergent changes (8.3%), while three studies recorded initial planned changes, followed by emergent changes (12.5%). Out of the 22 studies implementing planned changes, only four had stated the utilisation of structured change management models or methods, such as TeamSTEPPS or Kotter’s 8 Steps (13, 14, 17, 29).

Within the first theme of organisational restructuring, most changes were planned; for instance, the deliberate integration of mental health and social care services (36) and the merger of ICU units (30). Similarly, most of the changes under operational changes were planned, such as the implementation of a systematic crew resource management (CRM) in hospitals and universities (34). However, some changes grouped under this theme had both planned and emergent elements. For example, Ballangrud et al. (17) described how the implementation process of a planned TeamSTEPPS programme required emergent adaptations, such as changes in the composition of the management group. The third theme, adaptive response and capacity development, showed the most variability in the approach to change. While competency and capacity enhancement initiatives, such as structured training programmes, were typically planned (17), external responses and adaptations, particularly in studies documenting responses to COVID-19 (16, 18) and policy changes (23), were often undertaken according to immediate challenges.

The connection between the nature of the changes and teamwork outcomes exhibited mixed results. In terms of planned changes, positive teamwork outcomes were described in some cases, such as improved cohesion and a stronger bond between members after a care redesign (29); however, in other studies, negative outcomes were observed despite the planned nature of the changes. For example, during the amalgamation of two hospitals, teamwork was disrupted, as there was confusion regarding roles and responsibilities, resulting in instability within the team (25). Surprisingly, emergent changes, such as those implemented during the COVID-19 pandemic, produced some good outcomes by encouraging greater communication and support among workers (18). Still, teamwork suffered as the pandemic went on, and team members became more uncomfortable working together.

## Discussion

This systematic review synthesised the results from 24 studies and identified three key areas of organisational changes—organisational restructuring, operational changes, and adaptive response and capacity development—observed in healthcare settings. These changes potentially affect teamwork by influencing the team process, thus causing further deviations in teamwork outcomes, both of which were vital themes emerging from the examination of the impact of organisational changes on teamwork.

A key finding of this systematic review was that organisational changes, either planned, emergent, or both, resulted in complex and mixed effects on teamwork. Notably, deliberate and planned changes did not necessarily yield positive outcomes, and emergent and unplanned changes did not necessarily result in negative ones. This was evident in the studies included in the present systematic review. While certain planned changes resulted in improved collaboration and team functioning, others were associated with disrupted team dynamics and decreased performance. These findings indicate that the success of even planned changes aimed at improving teamwork outcomes largely depends on the implementation approach, situational context, and factors. While many organisations implemented changes, such as enhanced communication protocols and leadership structures, intended to promote team processes, the resultant impact on team outcomes was not always positive (14, 17, 32, 33). This discrepancy between process improvements and outcomes underscores the need for a more nuanced approach to understanding the influence of organisational changes on teamwork outcomes.

Improving teamwork outcomes or team effectiveness can be quite challenging due to the many contextual factors that require consideration; hence, understanding these specific factors is crucial before implementing any changes in an organisation. The input-process-output (IPO) model of team effectiveness provides a useful framework for understanding these complex relationships (37). In this model, “inputs” constitute the antecedent conditions for group activity; this includes environmental factors representing the broader context within which groups operate, such as organisational structure and culture (38). According to this model, the three themes of organisational changes identified in this review, namely organisational restructuring, operational changes, and adaptive response and capacity development, function as input variables that trigger further changes in team dynamics. By definition, the “processes” component functions as a mediating mechanism that transforms inputs into outputs; that is, it represents interactions among team members. This encompasses the theme of “team process” identified in this review, which includes the subthemes of leadership, decision-making, communication, and task organisation. Finally, “outputs” refer to the results of collective activity that are valued by the team or the organisation. Indicators of team effectiveness have generally been categorised into two groups, performance and member reactions, which correspond to the subthemes of teamwork outcomes identified in the current review.

The IPO model further clarifies why similar organisational changes can lead to different outcomes across settings, as the relationship between inputs and outputs is mediated by team processes that are essentially context-dependent. However, the model has been criticised for its assumption that group functioning is static and progresses linearly from inputs to outputs (39). The present review challenges this assumption in several ways. First, the changes seen in communication practices as a result of organisational reforms because of COVID-19 restrictions were dynamic (18). Furthermore, this review highlighted the presence of feedback loops that may not be readily apparent in the traditional IPO model; for example, team performance outcomes can become inputs for subsequent changes in team processes and states (17). Eventually, the traditional IPO model was deemed insufficient, and more advanced models and frameworks have emerged since then that perceive teams as dynamic and complex systems.

Mathieu et al. (40) mapped multiple team components that interact with each other to yield effectiveness and combined them into a framework comprising three main facets of teams: structural features, mediating mechanisms, and compositional features; some parts of these three facets overlap with each other. Rather than viewing organisational changes as discrete inputs that direct team processes and outcomes, the overlapping nature of the framework’s domains presents a more accurate representation of how structural changes, such as service integration, coexist and evolve with team processes. Instead of a linear cause-and-effect relationship, this framework helps to rationalise why similar organisational changes often lead to different consequences across different settings because of the interaction of these facets. It also helps explain the various impacts of organisational changes on teamwork seen across the included studies. The structural features of the framework proposed by Mathieu et al. (40) include organisational structure, team composition, and resources. Changes like mergers or structural organisations would alter these features, whereas compositional features, such as team members’ abilities and diversity, can also be affected by organisational changes, such as workforce reduction or new team compositions. This ultimately affects how teams interact effectively and adapt to individual performance, which is otherwise directly affected by sociodemographic differences (41). Furthermore, changes in decision-making processes, which form an important part of mediating mechanisms, can negatively impact the team’s adaptability and outcomes.

Among the studies included in this review, only four explicitly stated the application of recognised “change management frameworks” before implementing organisational modifications. Consistent with previous research, these studies predominantly demonstrated successful change implementation, resulting in primarily positive outcomes and favourable impacts on teamwork (42). Some negative outcomes were still observed, which may be mitigated through sustained effort and intervention from management. Implementing organisational changes, whether substantial or minor, presents significant challenges for healthcare administrators, particularly in terms of gaining acceptance from both individuals and teams (43). Consequently, using established change management frameworks can provide a strategic foundation for ensuring successful transformation within healthcare settings. A few examples of such frameworks include Kotter’s 8 Steps for Leading Change (44), Lewin’s 3 Stages of Change (45), and Prosci’s ADKAR Steps for Individual Change (43). Healthcare management professionals must acquire comprehensive knowledge of these management frameworks to ensure that change implementation is done in a systematic manner with successful outcomes.

### Limitations

This review has several limitations that should be considered when interpreting its findings. Most of the included studies (*n* = 23) were conducted in high-income countries, with only one study from an upper-middle-income country (South Africa). This limits the generalisability of the findings to healthcare settings in low- and middle-income countries where resource constraints and organisational structures pose significant challenges to care delivery. Furthermore, the majority of the studies were not longitudinal or cohort studies in which the same participants were followed up for the long term. Other studies conducted surveys pre- and post-organisational changes; however, since different participants were enrolled in the two surveys, the data analysis could not accurately assess the impact of organisational changes on teamwork. Finally, heterogeneity in the measurement of teamwork outcomes across studies obstructed direct comparisons between various types of organisational changes.

## Conclusion

This review qualitatively synthesised evidence from 24 studies to investigate the effects of organisational changes on teamwork within healthcare settings. Three primary themes were identified—organisational restructuring, operational changes, and adaptive response and capacity development—each associated with specific subthemes that encapsulate the diverse approaches to implementing changes at the organisational level. The findings reveal that organisational changes, whether planned or emergent, produce complex and context-dependent effects on teamwork. Planned changes do not always guarantee positive outcomes, while emergent changes can sometimes produce beneficial adaptations.

To maximise the effectiveness of organisational changes, healthcare leaders should utilise well-established change management frameworks that consider both contextual factors and interpersonal relationships. Further research is warranted, especially long-term studies and comprehensive data from low- and middle-income nations, to facilitate the development of robust and high-performing healthcare teams.

## Supplementary Material

**Table A:** Search strings

Database and date of search	Search string	No of hits
PubMed (25 Oct 2024)	(((“organizational change”[All Fields] OR “organizational structure”[All Fields] OR “organizational restructuring”[All Fields] OR “organizational redesign”[All Fields] OR “service integration”[All Fields] OR “organizational transformation”[All Fields] OR “restructuring”[All Fields] OR “reorganization”[All Fields] OR “mergers”[All Fields])) AND (“team effectiveness”[All Fields] OR “team performance”[All Fields] OR “teamwork”[All Fields] OR “team collaboration”[All Fields] OR “interprofessional collaboration”[All Fields] OR “multidisciplinary team”[All Fields] OR “interdisciplinary team”[All Fields]) AND (“healthcare”[All Fields] OR “health care”[All Fields] OR “hospital”[All Fields] OR “clinical”[All Fields] OR “medical”[All Fields] OR “health service”[All Fields]))	307
Web of Science (28 Oct 2024)	TS=((“organi?ational change” OR “organi?ational structure” OR “organi?ational restructuring” OR “organi?ational redesign” OR “service integration” OR “organizational transformation” OR restructuring OR reorganization OR mergers) AND (“team effectiveness” OR “team performance” OR teamwork OR “team collaboration” OR “interprofessional collaboration” OR “multidisciplinary team” OR “interdisciplinary team”) AND (healthcare OR “health care” OR hospital OR clinical OR medical OR “health service*”))	388
Scopus (28 Oct 2024)	TITLE-ABS-KEY ((“organi?ational change” OR “organi?ational structure” OR “organi?ational restructuring” OR “organi?ational redesign” OR “service integration” OR “organizational transformation” OR restructuring OR reorganization OR mergers) AND (“team effectiveness” OR “team performance” OR teamwork OR “team collaboration” OR “interprofessional collaboration” OR “multidisciplinary team” OR “interdisciplinary team”) AND (healthcare OR “health care” OR hospital OR clinical OR medical OR “health service*”))	977
ProQuest (28 Oct 2024)	NOFT((“organi?ational change” OR “organi?ational structure” OR “organi?ational restructuring” OR “organi?ational redesign” OR “service integration” OR “organizational transformation” OR restructuring OR reorganization OR mergers) AND (“team effectiveness” OR “team performance” OR teamwork OR “team collaboration” OR “interprofessional collaboration” OR “multidisciplinary team” OR “interdisciplinary team”) AND (healthcare OR “health care” OR hospital OR clinical OR medical OR “health service*”))	288

## Figures and Tables

**Table 1:** Summary of the characteristics and findings of the included studies (*n* = 24)

Author(s) (year) Country	Study population	Study design	Organisational changes	Type of changes	Teamwork outcomes	Quality of study
Gulliver et al. (2003) (36) England	Staff involved in the provision of adult mental health services	Cross-sectional	Integration of mental health and social care; teams were merged and new management structures were introduced	Planned	Integration strengthened collaboration but caused role confusion initially.	***
Cummings et al. (2005) (35) Canada	Registered nurses (RNs) working in acute care hospitals in Alberta	Cross-sectional	Hospital restructuring, leading to layoffs and reorganisation of nursing units	Planned	Reduced nursing collaboration resulting from unmet care needs.	****
Huby et al. (2008) (20) UK	Four general practices	Qualitative	General medical services contract reformed to allow centralised decision-making, expanded roles, and created hierarchical structures	Planned, followed by emergent	Centralised decision-making altered power dynamics and reduced communication.	*****
Mash et al. (2008) (13) South Africa	Primary health care practitioners in a few practices	Mixed-methods	Creation of practice teams, fostering collaboration through regular meetings, and introduction of participatory leadership	Planned	Participatory leadership improved cohesion, trust, and team functionality.	****
Gotlib Conn et al. (2010) (14) Canada	Members of a family health team in a primary care practice	Qualitative	Appreciative inquiry sessions improved communication and collaboration in family health teams	Planned	Incremental gains in communication and role clarity, but silos persisted.	*****
Allen et al. (2010) (25) Australia	Maternity health professionals working in maternity service of two public hospitals	Mixed-methods	Organisational restructuring, amalgamation of hospitals, and introduction of centralised systems	Planned	Centralisation reduced local ownership and weakened trust.	**
Grace et al. (2016) (33) US	Primary care physicians and staff across 33 practices	Cohort	Care team redesign with nurse managers and health coaches, support provided by training and workshops	Planned	No significant improvement in team climate or functionality.	*****
Maun et al. (2014) (19) Sweden	HCW from a primary care centre	Qualitative	Introduction of a patient-sorting system, streamlined workflows and resource allocation	Planned	Improved interprofessional communication and conflict resolution.	*****
Rajan et al. (2015) (24) European countries	Researchers, clinicians, nurses, and managers from eight cancer centres in Europe	Qualitative	Participation in European cancer accreditation improved data integration and multidisciplinary group (MDT) trust	Planned	Improved MDT communication, trust, and uniformity in processes.	*****
Vifladt et al. (2016) (30) Norway	RNs working in the ICU	Cross-sectional	ICU restructuring to merge units; formation of MIX-ICUs with new team dynamics	Planned	Decreased teamwork culture and cooperation.	****
Nutt and Keville (2016) (21) UK	Clinical psychologists working in adult community mental health teams	Qualitative	National Health Service restructuring reduced collaboration due to strained relationships and resource constraints	Planned	Reduced collaboration, reflective time, and communication.	*****
Martinussen et al. (2017) (31) Norway	All employees working in the Child and Family Unit of a municipal hospital	Cohort study	Reorganisation of services into a Child and Family Unit to enhance collaboration and service quality	Planned	Enhanced collaboration, reduced conflicts, and improved service quality.	****
Rodriquez (2017) (22) US	Staff working in the ICU of an academic medical centre	Qualitative	Managerial changes, increased ICU capacity, and reduced staff, focusing on workforce flexibility	Planned	Reduced resources and team identity, increased workload, and hindered care coordination.	*****
Hefner et al. (2017) (34) US	Primary care teams from an academic medical centre	Cross-sectional	CRM training to improve teamwork and safety culture	Planned	CRM training improved teamwork dimensions of patient safety culture.	****
Clay-Williams et al. (2018) (26) Australia	Staff responsible for the implementation of a merger in a tertiary care hospital and clinical staff affected by the merger in the medical ward	Mixed-method study using a convergent parallel approach	Introduction of structured interdisciplinary rounds to enhance communication and workflow	Planned	Enhanced communication, trust, and inclusivity despite interdepartmental strains.	****
Bentley et al. (2018) (27) Australia	Public and private primary care services	Sequential mixed-method design	Budget cuts, policy shifts, and relocations	Planned	Reduced informal collaboration and disrupted interprofessional team cohesion.	****
MacKinnon et al. (2018) (15) Canada	RNs and practical nurses (PLNs) from two community hospitals	Qualitative	Care Delivery Model replaced RN-centric care with team-based roles and structured communication	Planned	Structured communication tools fostered coordination but increased tensions.	*****
Pandhi et al. (2018) (29) US	HCW from primary care teams at a large academic healthcare system	Mixed-methods	Microsystems approach in primary care, including team coaching and structured improvement processes	Planned	Better relationships, problem-solving, and meeting effectiveness despite instability.	****
Cain (2019) (23) US	Hospice care staff members	Qualitative	Medicare-driven cost-cutting and staff reductions; reinforcing medical over interdisciplinary logics	Planned, followed by emergent	Weakening of interdisciplinary teams and worsening collaboration.	*****
Milton et al. (2020) (32) Sweden	Staff working in the emergency department of a university hospital	Cross-sectional	Emergency department improvements through team training, ethical workshops, and optimised spaces	Planned	Minimal improvement in teamwork climate scores.	****
Ballangrud et al. (2021) (17) Norway	Healthcare professionals from the surgical ward of a hospital trust	Qualitative	TeamSTEPPS training programme to improve interprofessional collaboration with simulation exercises	Planned, followed by emergent	Improved cohesion and situational awareness, though barriers persisted.	*****
Pulido-Fuentes et al. (2022) (18) Spain	Primary care workers in two public healthcare services	Qualitative	COVID-19-driven role restructuring in primary care	Emergent	Initial improvement in collaboration; later, teamwork was disrupted by pandemic fatigue.	*****
Milton et al. (2023) (28) Sweden	Healthcare practitioners working in the emergency department of a university hospital	Mixed-methods study with pre-and post-intervention assessment	Implementation of interprofessional teamwork, rapid emergency triage and treatment system-based triage, simulation training, and structural improvements	Planned	Improved team communication and reduced interruptions.	****
Rogers et al. (2023) (16) Ireland	Multidisciplinary team members from a diverse range of professions within the Irish health system	Qualitative	COVID-19 pandemic-induced MDT changes, including virtual meetings and modified care models	Emergent	Shifted MDT dynamics increased collaboration, but staff turnover impacted outcomes.	*****

CRM = crew resource management; ICU = intensive care unit; UK = United Kingdom; US = United States; HCW = healthcare workers; MDT = multidisciplinary team; MIX-ICU = mixed intensive care unit; TeamSTEPPS = team strategies and tools to enhance performance and patient safety

**Table 2:** Quality appraisal of included studies using MMAT

Author(s) (year)	Criteria from the MMAT																								
	1.1	1.2	1.3	1.4	1.5	2.1	2.2	2.3	2.4	2.5	3.1	3.2	3.3	3.4	3.5	4.1	4.2	4.3	4.4	4.5	5.1	5.2	5.3	5.4	5.5
Gulliver et al. (2003) (36)																1	1	1	0	0					
Cummings et al. (2005) (35)																1	1	1	0	1					
Huby et al. (2008) (20)	1	1	1	1	1																				
Mash et al. (2008) (13)	1	1	1	1	1											1	1	1	0	1	1	1	1	0	1
Gotlib Conn et al. (2010) (14)	1	1	1	1	1																				
Allen et al. (2010) (25)	1	1	1	1	1											1	0	1	0	0	1	1	0	0	1
Grace et al. (2016) (33)						1	1	1	1	1	1	1	1	1	1										
Maun et al. (2014) (19)	1	1	1	1	1																				
Rajan et al. (2015) (24)	1	1	1	1	1																				
Vifladt et al. (2016) (30)																1	1	1	0	1					
Nutt and Keville (2016) (21)	1	1	1	1	1																				
Martinussen et al. (2017) (31)						1	1	1	0	1	1	1	1	0	1										
Rodriquez (2017) (22)	1	1	1	1	1																				
Hefner et al. (2017) (34)																1	1	1	0	1					
Clay-Williams et al. (2018) (26)	1	1	1	1	1											1	1	1	1	1	1	1	1	0	1
Bentley et al. (2018) (27)	1	1	1	1	1											1	1	1	0	1	1	1	1	0	1
MacKinnon et al. (2018) (15)	1	1	1	1	1																				
Pandhi et al. (2018) (29)	1	1	1	1	1	1	1	1	0	1	1	1	1	0	1						1	1	1	1	1
Cain (2019) (23)	1	1	1	1	1																				
Milton et al. (2020) (32)																1	1	1	0	1					
Ballangrud et al. (2021) (17)	1	1	1	1	1																				
Pulido-Fuentes et al. (2022) (18)	1	1	1	1	1																				
Milton et al. (2023) (28)	1	1	1	1	1	1	1	1	0	1	1	1	1	0	1						1	1	1	1	1
Rogers et al. (2023) (16)	1	1	1	1	1																				

MMAT = mixed-methods appraisal tool

**Table 3:** Themes of organisational changes

Author(s) (year)	Organisational restructuring			Operational changes			Adaptive response and capacity development		
	Service integration and merger	Workforce reorganisation	Physical and environmental changes	Process and technology changes	Governance and management changes	Changes in working methods and protocols	Capacity and skills enhancement	Collaborative practice and culture	Response and adaptation to external pressure
Gulliver et al. (2003) (36)	√				√			√	
Cummings et al. (2005) (35)		√							
Huby et al. (2008) (20)		√			√				
Mash et al. (2008) (13)			√		√			√	
Gotlib Conn et al. (2010) (14)							√		
Allen et al. (2010) (25)	√			√					
Grace et al. (2016) (33)		√							
Maun et al. (2014) (19)				√				√	
Rajan et al. (2015) (24)									√
Vifladt et al. (2016) (30)	√								
Nutt and Keville (2016) (21)									√
Martinussen et al. (2017) (31)	√								
Rodriquez (2017) (22)		√			√				
Hefner et al. (2017) (34)						√	√		
Clay-Williams et al. (2018) (26)	√			√					
Bentley et al. (2018) (27)			√	√					√
MacKinnon et al. (2018) (15)		√				√			
Pandhi et al. (2018) (29)						√	√		
Cain (2019) (23)									√
Milton et al. (2020) (32)			√				√		
Ballangrud et al. (2021) (17)							√	√	
Pulido-Fuentes et al. (2022) (18)									√
Milton et al. (2023) (28)			√			√		√	
Rogers et al. (2023) (16)		√		√					√

**Table 4:** Themes of the impact of organisational changes on teamwork

Author(s) (year)	Team processes		Teamwork outcomes	
	Leadership and decision-making	Communication and task organisation	Team performance	Impact on the members of the team
Gulliver et al. (2003) (36)				√
Cummings et al. (2005) (35)	√		√	
Huby et al. (2008) (20)	√			
Mash et al. (2008) (13)	√		√	
Gotlib Conn et al. (2010) (14)		√		
Allen et al. (2010) (25)	√		√	
Grace et al. (2016) (33)			√	
Maun et al. (2014) (19)		√		√
Rajan et al. (2015) (24)		√	√	
Vifladt et al. (2016) (30)	√		√	
Nutt and Keville (2016) (21)		√		√
Martinussen et al. (2017) (31)			√	
Rodriquez (2017) (22)			√	
Hefner et al. (2017) (34)		√		
Clay-Williams et al. (2018) (26)	√			
Bentley et al. (2018) (27)		√	√	
MacKinnon et al. (2018) (15)		√		
Pandhi et al. (2018) (29)			√	
Cain (2019) (23)				√
Milton et al. (2020) (32)		√		
Ballangrud et al. (2021) (17)			√	
Pulido-Fuentes et al. (2022) (18)		√	√	
Milton et al. (2023) (28)		√		
Rogers et al. (2023) (16)		√		
